# Effect of Returning University Students on COVID-19 Infections in England, 2020

**DOI:** 10.3201/eid2807.212332

**Published:** 2022-07

**Authors:** David Leeman, Joe Flannagan, Dimple Chudasama, Kyle Dack, Charlotte Anderson, Gavin Dabrera, Theresa Lamagni

**Affiliations:** UK Health Security Agency, London, UK

**Keywords:** COVID-19, 2019 novel coronavirus disease, coronavirus disease, severe acute respiratory syndrome coronavirus 2, SARS-CoV-2, viruses, respiratory infections, zoonoses, universities, students, England

## Abstract

Each September in England, ≈1 million students relocate to study at universities. To determine COVID-19 cases and outbreaks among university students after their return to university during the COVID pandemic in September 2020, we identified students with COVID-19 (student case-patients) by reviewing contact tracing records identifying attendance at university and residence in student accommodations identified by matching case-patients’ residential addresses with national property databases. We determined COVID-19 rates in towns/cities with and without a university campus. We identified 53,430 student case-patients during September 1–December 31, 2020, which accounted for 2.7% of all cases during this period. Student case-patients increased rapidly after the start of the term, driven initially by cases and outbreaks in student accommodations. Case rates among students 18–23 years of age doubled at the start of term in towns with universities. Our findings highlight the need for face-to-face and control measures to reduce virus transmission.

COVID-19 cases in England first peaked in April 2020, after national lockdown measures were introduced on March 26, 2020; cases decreased in June 2020 and remained relatively low throughout the summer. Starting at the end of August, cases increased, especially during October and November (*1*). Because September is the beginning of the academic year in the United Kingdom, this growth coincided with the annual mass migration of university students across the country.

Approximately 2.5 million students study at higher education institutions in the United Kingdom (*2*), accounting for ≈3% of the UK population; 2 million study at universities in England. In the 2019–20 academic year, ≈1.1 million full-time students lived in accommodations other than their normal residence (their own or their parents’/guardians’) (*3*). Concerns were raised over the return of university students for face-to-face learning in the 2020 autumn term. Some institutions decided to keep learning online, but overall, the government advised universities to encourage in-person return. Immediately after the start of the term, COVID-19 outbreaks associated with universities were identified and received substantial media attention. On October 12, the government stated that 9,000 COVID-19 cases had been identified among students in the previous week and that 1 university (Nottingham) accounted for 1,510 of these cases (*4*).

Several projects have been undertaken to learn more about transmission of SARS-CoV-2 within educational establishments (*5*) and infection rates among the school-age population (*6*). We used national testing and contact tracing data linked to property classifications to describe SARS-CoV-2 infections among those reporting attendance at a university and those living in student accommodations.

The UK Health Security Agency has legal permission, provided by Regulation 3 of The Health Service (Control of Patient Information) Regulations 2002, to process patient confidential information for national surveillance of communicable diseases. Thus, individual patient consent was not required.

## Methods

### Data Collection

We extracted cases from the second-generation surveillance system (*7*). All test-positive cases of COVID-19 are notifiable through reporting to the second-generation surveillance system, including positive results from lateral flow devices. During the study period, routine testing of asymptomatic persons was not yet available, so reported case-patients were predominately symptomatic. Mass testing for asymptomatic students was introduced at the end of November 2020, when students were asked to complete testing before returning home (*8*). Routine testing for asymptomatic students was not introduced until the spring term of 2021 (*9*).

As part of routine contact tracing, to identify presymptomatic contacts and potential sources of infection, persons with positive test results were asked about their events and activities of the 7 days before symptom onset (or test date), including whether they had attended an education setting, up to the time of contact tracing. To identify all case-patients reporting attendance at a university, those with positive test results were linked to exposure data in National Health Service (NHS) Test and Trace (https://www.gov.uk/guidance/nhs-test-and-trace-how-it-works). On October 23, 2020, the standard questions changed to further differentiate between attending or working at a university.

### Data Linkage and Assignment

Case-patient and contact tracing data were linked by the specimen number of the positive test. Case-patients that did not link by specimen number were linked by NHS number and date of birth.

We identified accommodation type by matching case-patients’ full addresses to the reference database Ordnance Survey Address Base Premium (*10*). This database provided each address with a unique property reference number (UPRN); a basic land and property unit (BLPU) class; and where available, a parent UPRN enabling us to map case-patients against a specific residential location. Parent UPRNs exist for properties that may have multiple subproperties within (e.g., a block of flats for which the parent UPRN identifies the entire block and individual UPRNs are assigned to the individual flats).

To determine whether student case-patients affected case rates among the wider population, we compared age-specific case rates between university and nonuniversity towns throughout the autumn term by using Office for National Statistics (ONS) 2019 midyear population estimates by age (*11*). ONS midyear estimates use census data to provide official population estimates and count students at their term-time addresses (*12*). Towns and cities were identified by using the ONS major towns and cities dataset, which includes towns or cities with a resident or workday population of >75,000 (*13*). Cases were matched to these municipalities by the lower superoutput area associated with their postal code of residence. We matched universities to towns by using the registered address as recorded against their learning provider reference number (*14*). We manually reviewed remaining towns to check for satellite campuses or other higher education institutions (some higher education institutions are not registered providers and have their qualifications granted by another institution and therefore have no learning provider reference number). We excluded from analysis towns with only satellite campuses.

We identified towns with no university and >60 minutes travel by public transport (based on Google Maps journey planner) from the nearest university campus and matched them to a university town according to region and population density within 20%. Because we found multiple matches, with either nonuniversity towns matching to multiple university towns or vice versa, we created a loop by randomly selecting matched towns until each pair was unique with no duplicated towns.

### Definitions

We defined case-patients as all persons with positive test results reported to the second-generation surveillance system with an earliest reported specimen date of September 1–December 31, 2020. Student accommodation was defined as 1 of the following: properties with a BLPU classification of higher education or university; properties with a classification of college, in which all case-patients were >18 years of age; properties with a classification of residential education, for which the address included any of the terms university, hall of residence, halls of residence, student accommodation; properties with a classification of residential education and for which *>*90% of case-patients were >18 years of age; properties classified as parent shell or property shell, for which the address included any of the terms university, student, hall of residence, halls of residence; or properties with a classification of college, other educational establishment, residential education, parent shell, or property shell, from which >5 case-patients reported attendance at university to contact tracers. Students were defined as case-patients if they either resided in student accommodation premises as defined above or reported attendance at a university to NHS Test and Trace. Outbreaks were defined as >2 cases at the same residence (determined by UPRN), within a recurring 14-day period. Properties could have multiple outbreaks recorded if further cases were identified >14 days past a previous outbreak. University towns were defined as any town or city in England meeting the ONS definition of major towns and cities with a higher education campus. Nonuniversity towns were defined as any town or city in England meeting the ONS definition of major towns and cities with no higher education campus and requiring >60 minutes travel from the nearest campus by public transport.

### Analysis

We described case-patients according to demographics and accommodation type by collapsing BLPU classifications into 4 main groups: residential accommodation (i.e., houses or flats/apartments), student accommodations (as defined previously), registered houses of multiple occupancy (HMO), and other. We described outbreaks involving students according to size, duration of time between the first and last case, and property type. We compared these outbreaks with outbreaks involving no identified students. We compared university and nonuniversity towns by calculating and plotting rates for the total population, for the population 18–23 years of age, and the total population minus those 18–23 years of age.

## Results

### Classification of Student Case-Patients and Student Accommodations

During September 1–December 31, 2020, a total of 1,999,180 cases of COVID-19 were reported in England. Contact tracing with NHS Test and Trace was completed for 1,648,220 (82.4%), among which attendance at a university was reported by 39,032 (2.4%).

Among all 1,999,180 case-patients, 19,901 (1%) resided in a property classified as student accommodation (1,820 UPRNs met the definition of student accommodation; Appendix Figure 1). A total of 53,430 (2.7%) case-patients met the definition of student on the basis of residence or information relayed to contact tracers; these students are hereafter referred to as student case-patients.

Most (33,529 [85.9%]) case-patients who reported university attendance to contact tracers did not live in student accommodations. This percentage decreased to 73% (4,844) of 6,632 students 18 years of age who attended university. For all case-patients living in student accommodations, the median age was 19 (interquartile range [IQR] 18–20]) years, which was slightly younger than the age of student case-patients living in other types of accommodation (median age 20 [IQR 19–22]) years. This age profile reflects the common nature of student accommodations being used by first-year students who then move on to privately rented accommodations.

### Description of Student Case-Patients

**Figure 1:**
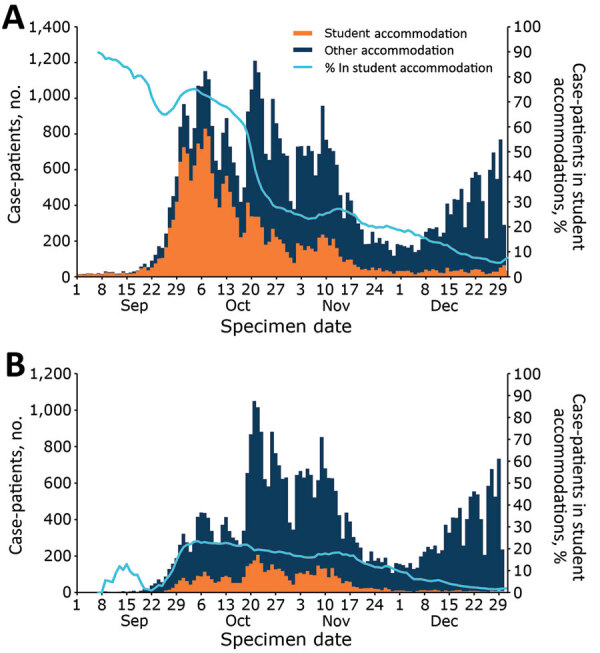
Student COVID-19 case-patients, by specimen date and accommodation type, England, September 1–December 31, 2020.

The median age for student case-patients was 20 (IQR 19–22) years, 17 years younger than the median age for all case-patients. A slightly higher proportion of student case-patients than of case-patients were female (57.1% vs. 53.2%) (Table 1). Case numbers among students and the proportion of all cases they represented increased rapidly during the start of the university term from 0.7% (117/17,508) in the first week of September to 7.8% (6,709/85,929) in the first week of October. At the beginning of the term, ≈60%–70% of student case-patients resided in student accommodations, and although the number of student case-patients remained high until mid-November, the proportion in student accommodations had dropped to 20%–30%. When we considered only case-patients who reported attendance at a university, the trend differed with a smaller increase in cases at the start of the term and a lower proportion residing in student accommodations (Figure 1). The peak of infections for all student case-patients was reached quickly after the start of term in the first week of October but peaked around 2 weeks later among those specifically reporting attendance at a university.

**Table 1:** Characteristics of COVID-19 case-patients, by university classification, England, September 1–December 31, 2020*

Characteristic	No. (%) students			
	Attending university, n = 39,032†	Residing in student accommodation, n = 19,901‡	Total student case-patients, n = 53,430§	Total case-patients, n = 1,999,180¶
Sex				
F	22,517 (57.7)	11,109 (55.8)	30,526 (57.1)	1,063,624 (53.2)
M	15,833 (40.6)	8,555 (43.0)	22,042 (41.3)	918,902 (46.0)
Unknown	682 (1.7)	237 (1.2)	862 (1.6)	16,654 (0.8)
Ethnicity				
Asian/Asian British	6,479 (16.6)	1,847 (9.3)	7,775 (14.6)	285,562 (14.3)
Black/Black British	2,060 (5.3)	758 (3.8)	2,566 (4.8)	78,529 (3.9)
Mixed	1,519 (3.9)	800 (4.0)	2,085 (3.9)	45,762 (2.3)
Other	991 (2.5)	329 (1.7)	1,207 (2.3)	37,166 (1.9)
Unknown	1,744 (4.5)	1,357 (6.8)	2,790 (5.2)	75,857 (3.8)
White	26,239 (67.2)	14,810 (74.4)	37,007 (69.3)	1,476,304 (73.8)
Region				
East Midlands	4,487 (11.5)	3,884 (19.5)	7,292 (13.6)	166,352 (8.3)
East of England	3,160 (8.1)	1,184 (5.9)	3,854 (7.2)	199,341 (10.0)
London	7,136 (18.3)	1,419 (7.1)	8,056 (15.1)	378,483 (18.9)
North East	1,843 (4.7)	1,808 (9.1)	3,411 (6.4)	109,891 (5.5)
North West	4,211 (10.8)	2,395 (12.0)	6,265 (11.7)	326,296 (16.3)
South East	5,694 (14.6)	1,788 (9.0)	6,794 (12.7)	261,810 (13.1)
South West	4,394 (11.3)	2,801 (14.1)	6,194 (11.6)	107,119 (5.4)
West Midlands	3,836 (9.8)	1,535 (7.7)	4,833 (9.0)	208,755 (10.4)
Yorkshire and Humber	3,806 (9.8)	3,067 (15.4)	6,251 (11.7)	224,888 (11.2)
Unknown	465 (1.2)	20 (0.1)	480 (0.9)	16,245 (0.8)
Accommodation type				
Student accommodation	5,503 (14.1)	19,901 (100)	19,901 (37.2)	19,901 (1.0)
Detached house	6,446 (16.5)		6,446 (12.1)	341,391 (17.1)
Semidetached house	7,142 (18.3)	0	7,142 (13.4)	552,024 (27.6)
Terraced house	9,347 (23.9)	0	9,347 (17.5)	589,178 (29.5)
Flat	4,193 (10.7)	0	4,193 (7.8)	246,493 (12.3)
HMO	1,617 (4.1)	0	1,617 (3.0)	9,838 (0.5)
Property shell	70 (1.8)	0	700 (1.3)	18,554 (0.9)
Other	4,08 (10.5)	0	4,084 (7.6)	221,801 (11.1)
Deaths#	15 0	14 (0.1)	28 (0.1)	53,648 (2.7)
Cases in outbreak	17,553 (45.0)	14,375 (72.2)	27,90 (52.2)	964,902 (48.3)

*HMO, houses of multiple occupancy. †Median age 20 y. ‡Median age 19 y. §Median age 20 y. Because data for case-patients can appear in both the attending university and student accommodation columns, the total is less than the sum of these 2 columns. ¶Median age 37 y. #Deaths recorded within 28 d of first positive test result.

We found a similar, but more pronounced, trend among the student-age population (18–23 years of age). Case numbers and rates increased substantially among this population from 11 cases/100,000 persons in this age range in England on September 1, 2020, to 99 cases/100,000 persons on October 1, 2020. Comparatively, the rate among the rest of the population increased from 3 to 13 cases/100,000 persons in the same period. By the end of September 2020, case-patients 18–23 years of age accounted for ≈30% of all cases in England, reaching a daily high of 44.1% (3,842/8,718) on September 29, 2020.

### Accommodation Types and Residential Outbreaks

**Figure 2:**
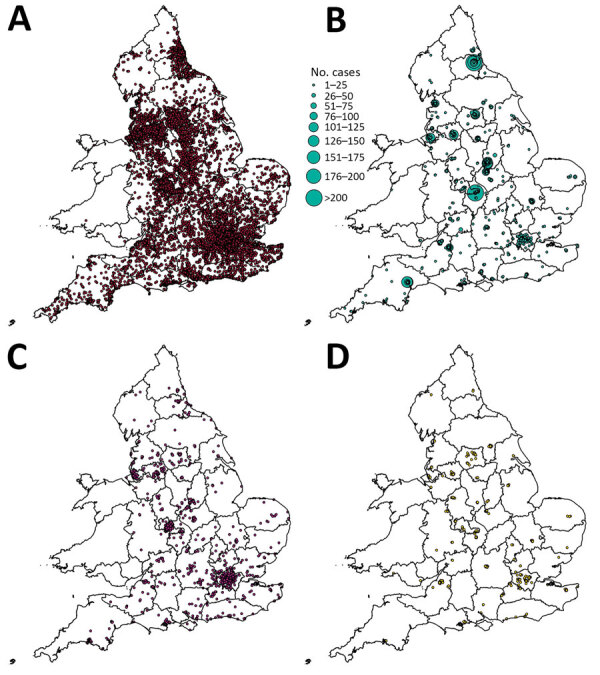
COVID-19 student case-patients, by location and property type, England, September 1–December 31, 2020. A) Private residences; B) student accommodations, showing rates of student cases; C) other accommodation type; D) houses of multiple occupancy.

Student case-patients were geographically dispersed across England, concentrated around major urban areas (Figure 2). When counted by upper tier local authority boundary, the highest number of student case-patients was in Nottingham (n = 3,021), >1,000 more than the second highest, who were in Sheffield (n = 1,976). Other areas with >1,500 case-patients were Manchester (n = 1,912), Bristol (n= 1,710), Leeds (n = 1,681), and Birmingham (n = 1,544). Most of the 53,450 student case-patients lived either in student accommodations (19,901 [37.2%]) or private residential properties such as houses or flats (27,128 [50.8%]). A smaller proportion (3%, n = 1.617) lived in HMOs, but this proportion was 6 times larger than that of all case-patients who lived in HMOs; 16.4% (1,617/9,838) of all case-patients who resided in HMOs were identified as students. The highest proportions of student case-patients living in student accommodations were in Nottingham (76.7%, n = 2,316/3,021), Sheffield (75.6%, n = 1,494/1,976), York (73.2%, n = 517/706), Coventry (71.2%, n = 679/954), and Newcastle (70.3%, n = 937/1,333). Nottingham had the highest number of case-patients living in student accommodations (n = 2,316).

Nearly half of all case-patients (48.3%, n = 964,902) were identified as being involved in 1 of 371,937 residential outbreaks. Just under 4% of these outbreaks included >1 student (3.6%, 13,572/371,937). Outbreaks that included >1 student had a median number of 3 (IQR 2–4) cases/outbreak, which was slightly more than that of all other outbreaks (median 2, IQR 2–3 cases) (Table 2). Outbreaks within student accommodations lasted a median of 6 (IQR 2–13) days, compared with 2 (IQR 0–5) days for all other residential settings.

**Table 2:** Characteristics of residence types involved with COVID-19 outbreaks, England, September 1–December 31, 2020*

Property type	No. clusters	Clusters including a student, no.	Clusters including a student, %	Outbreaks containing students			
				Median cluster size, no. cases	Cluster size range (IQR)	Med cluster duration d	Cluster duration range (IQR)
Detached	74,580	2,189	2.9	3	2–12 (2–4)	3	0–25 (1–5)
Semidetached	116,554	3,008	2.6	3	2–14 (2–4)	3	0–31 (1–6)
Terraced	119,327	3,967	3.3	3	2–9 (2–4)	3	0–25 (1–5)
Flat	40,509	1,440	3.6	2	2–15 (2–3)	2	0–31 (0–5)
HMO	1,402	423	30.2	2	2–20 (2–3)	2	0–26 (0–5)
Property shell, not defined	2,683	207	7.7	2	2–28 (2–3)	3	0–37 (1–7)
Student accommodation	1,917	1,917	100	3	2–229 (2–5)	6	0–75 (2–13)
Other	14,965	421	2.8	3	2–114 (2–4)	3	0–58 (1–7)
Total	371937	13,572	3.6	3	2–229 (2–4)	3	0–75 (1–6)

*HMO, houses of multiple occupancy; IQR, interquartile range.

**Figure 3:**
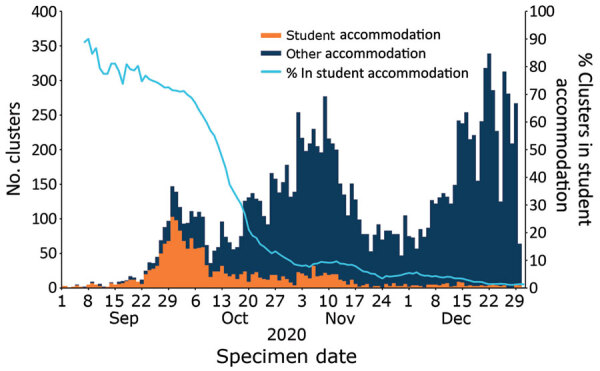
COVID-19 residential clusters involving >1 student, by specimen date of first case and accommodation type, England, September 1–December 31, 2020.

The number of residential outbreaks involving students after the start of the term increased immediately. This increase was largely driven by outbreaks within student accommodations; at the start of the term; ≈70% of outbreaks were in student accommodations. The initial increase was followed by 2 larger peaks, mainly in other residential settings (Figure 3). Those 2 peaks followed the trend for all residential outbreaks during this period; the number of outbreaks decreased after the introduction of national restrictions in November.

Overall, the proportion of outbreaks involving students increased from 0.7% (18/2,440) in the first week of September 2020 to 7.3% (519/7,149) in the final week of September 2020. This proportion decreased and remained at 3%–5% until the end of December, when it returned to 1%.

### Comparison between Towns

**Figure 4:**
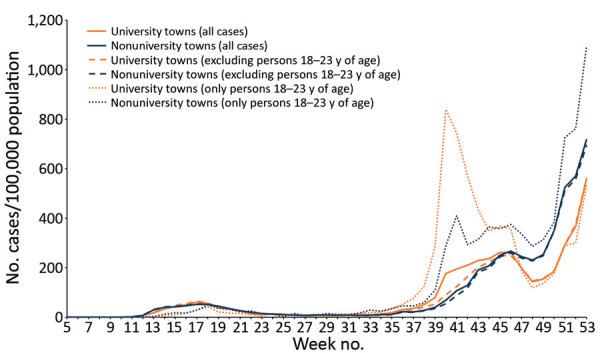
COVID-19 rates (cases/100,000 population) in selected university and nonuniversity towns, England, 2020.

We identified 20 towns (10 with universities and 10 without) for comparing COVID-19 incidence rates during the commencement of the autumn term (Appendix Table 1). Rates in university towns showed a clear uptick at the start of the term (week 39), and rates in nonuniversity towns increased with a more gradual slope. When split by age, this increase in rates among university towns was clearly being driven by those 18–23 years of age, for whom rates were double those seen in nonuniversity towns (Figure 4). However, as the rate increase in university towns slowed, the overall rate in nonuniversity towns caught up by week 46.

A second period of national restrictions was introduced on November 5; COVID-19 incidence rates decreased in the following weeks. The reduction was more pronounced in university towns; overall rates decreased only modestly in nonuniversity towns. The restrictions ended on December 2, after which rates in nonuniversity towns increased faster than in university towns. Furthermore, rates among persons 18–23 years of age in university towns were much closer to the overall rates, whereas in nonuniversity towns, rates remained higher. By week 51, the cumulative rate in nonuniversity towns had overtaken that of university towns; the following week, the cumulative rate in persons 18–23 years of age in nonuniversity towns also overtook that of rates for university towns (Appendix Figure 2).

## Discussion

Our novel approach for classifying COVID-19 case-patients as students on the basis of address classifications and contact tracing data showed a large increase in student case-patients immediately after the start of term. The increase was initially driven by students residing in student accommodations, followed by students living in other types of accommodations. Although case-patients in student accommodations initially peaked at the start of term, followed by some peaking at much lower levels, student case-patients in other types of accommodations peaked at high levels multiple times during the term. We hypothesize that this difference most likely resulted from the combination of stricter enforcement of control measures in university accommodations, where student self-isolation was enforced by campus security (*15*), and the smaller proportion of students living in student accommodations (*16*) resulting in fewer susceptible students after the initial peak.

Increased rates of COVID-19 cases spilled over into the broader student-age population; rates among persons 18–23 years of age in university towns were double that in nonuniversity towns. Rates were substantially affected by the start of national restrictions, after which rates were consistently higher in nonuniversity towns than in university towns. The combination of enforced control measures in student accommodations and national restrictions seems to have had a greater effect in university towns, but without those measures, high rates of transmission among the student-age population would probably have been sustained.

Other studies have similarly shown high case numbers and outbreaks among students and within student accommodations in the United Kingdom (*5*,*17*) and the United States (*18*,*19*), particularly when students return to campus and student accommodations. Less is known about the effect on the wider community around a campus (*20*; C.R.K. Arnold et al., unpub. data, https://www.medrxiv.org/content/10.1101/2021.02.17.21251942v5). Our study shows a deviation of rates at the start of term between university and nonuniversity towns. The effect on wider communities was limited, but rates among persons 18–23 years of age increased 2-fold more in towns with a university campus than in towns without. However, a recent study that used genomic data on university and nonuniversity cases associated with Cambridge University in Cambridge, UK, found limited evidence of transmission across the student and local populations (*21*).

Although routinely monitored severe outcomes, including hospitalizations and deaths, are less common among young adults, COVID-19 infections have substantial direct and indirect effects on young adults. Hospitalizations and deaths do occur among persons in this age group (*22*), and young adults who are hospitalized experience a range of adverse outcomes (*23*). COVID-19 has significantly negatively affected the mental health of young persons, particularly in relation to lockdowns and long periods of self-isolation (*24*). The common use of online teaching added to feelings of isolation and loneliness; >50% of students reported dissatisfaction with their social experiences during the autumn term (*5*). The full effect on student achievements and well-being as a result of these disruptions is unlikely to be fully appreciated for some time.

Information about transmission among this population remains limited, but the concentration of cases initially in student accommodations and the shared living arrangements reflect known transmission dynamics of SARS-CoV-2; household transmission is a significant source of infection (*25*). Less well known is the role of face-to-face teaching and in-person lectures with regard to virus transmission between households. The beginning of a term in UK universities is known as fresher’s week, when new students arrive in university towns a week before the start of teaching to meet other students and participate in a variety of organized and spontaneous social activities. This mixing of a large, susceptible population from across the country in crowded, enclosed spaces is likely to result in increased cases and poses the potential for more large outbreaks and disruption to teaching across higher-education providers. The 2021–22 academic year differed from the previous academic year in terms of testing and the introduction of COVID-19 vaccinations. By the start of the 2021–22 term, <50% of persons 18–24 years of age had received both vaccine doses (*26*), and case levels remained high throughout the summer and autumn across the entire population, particularly among children and young adults. However, the removal of restrictions and the emergence of the Omicron variant have made ascertaining the effects of student migration on transmission even more challenging.

Ordnance Survey data (https://www.ordnancesurvey.co.uk) to enrich COVID-19 data in England have been used to monitor cases and outbreaks in households (*27*) and within specific properties, including care homes and prisons. Although bespoke student accommodation is a regular feature of universities within England, these premises are not uniformly categorized or recorded; thus, we created a method for categorizing them. Specificity of the definitions used will have led to underestimation of case-patients residing in student accommodations.

By combining data from NHS Test and Trace, we were able to identify a sizable proportion of case-patients that we can confidently define as students. However, an unknown proportion of student case-patients either did not engage with contact tracers or did engage but had not physically attended their campus in the prior 7 days and thus not have been identified as student case-patients. Although using both data sources instead of either source independently enabled us to classify more student case-patients, using both sources favors detection of student case-patients in student accommodations because they can be identified by either method, whereas case-patients in other residential settings can be identified only by contact tracing data. As a result, we have probably overestimated the proportion of case-patients residing in student accommodations; however, because 33,529 student case-patients were identified in other types of accommodation, the effect of overestimation here is probably small.

The comparison between university and nonuniversity towns is limited by the potential for systematic differences between towns that have a university and those that do not. Socioeconomic deprivation and other demographics that affect COVID-19 rates (*28*) have not been accounted for when comparing these towns because of the limited number of nonuniversity towns in England. Therefore, despite a clear difference, we cannot state how much of the observed difference in rates results from the presence or absence of a university within these towns.

Our findings suggest that the annual mass migration of students and housing of large numbers in student accommodations is linked to large increases in SARS-CoV-2 transmission among this population, potentially contributing to large increases in cases in the wider population surrounding a campus. The desire for face-to-face teaching requires this migration because of the preponderance of students in England who study away from home. We therefore recommend further assessment of policy decisions advocating universities’ return to face-to-face teaching to ensure that the risks associated with a large increase in case numbers and outbreaks in this population are balanced against the risks associated with remote and online teaching.

Appendix Additional information on effect of returning university students on COVID-19 cases in England, 2020.

## References

[1] UK Government. Coronavirus (COVID-19) in the UK [cited 2021 Jan 18]. https://coronavirus.data.gov.uk

[2] Universities UK. Higher education in numbers [cited 2021 Jan 18]. https://www.universitiesuk.ac.uk/what-we-do/policy-and-research/publications/higher-education-facts-and-figures-2021

[3] Higher Education Statistics Agency. Where do HE students study? [cited 2021 Sept 14]. https://www.hesa.ac.uk/data-and-analysis/students/where-study

[4] Times Higher Education. COVID-19: UK confirms 9,000 student cases in past week [cited 2021 Jan 18]. https://www.timeshighereducation.com/news/covid-19-uk-confirms-9000-student-cases-past-week

[5] Office for National Statistics. How has coronavirus (COVID-19) spread among students in England? [cited 2021 Jan 19]. https://www.ons.gov.uk/peoplepopulationandcommunity/educationandchildcare/articles/howhascoronaviruscovid19spreadamongstudentsinengland/2020-12-21

[6] Mensah AA, Sinnathamby M, Zaidi A, Coughlan L, Simmons R, Ismail SA, et al. SARS-CoV-2 infections in children following the full re-opening of schools and the impact of national lockdown: Prospective, national observational cohort surveillance, July-December 2020, England. J Infect. 2021;82:67–74. 10.1016/j.jinf.2021.02.022 33639175 PMC7904496

[7] Clare T, Twohig KA, O’Connell A-M, Dabrera G. Timeliness and completeness of laboratory-based surveillance of COVID-19 cases in England. Public Health. 2021;194:163–6. 10.1016/j.puhe.2021.03.012 33945929 PMC8015423

[8] Government HM. COVID-19 winter plan [cited 2021 Oct 3]. https://assets.publishing.service.gov.uk/government/uploads/system/uploads/attachment_data/file/937529/COVID-19_Winter_Plan.pdf

[9] Gov.uk. All students offered testing on return to university [cited 2021 Oct 3]. https://www.gov.uk/government/news/all-students-offered-testing-on-return-to-university

[10] Chudasama DY, Milbourn H, Nsonwu O, Senyah F, Florence I, Cook B, et al. Penetration and impact of COVID-19 in long term care facilities in England: population surveillance study. Int J Epidemiol. 2021;Sep 1:dyab176. 10.1093/ije/dyab176 34999883

[11] Office for National Statistics. Lower layer Super Output Area population estimates (supporting information) [cited 2021 May 29]. https://www.ons.gov.uk/peoplepopulationandcommunity/populationandmigration/populationestimates/datasets/lowersuperoutputareamidyearpopulationestimates

[12] Office for National Statistics. Mid-year population estimates quality and methodology information [cited 2022 Mar 31]. https://www.ons.gov.uk/peoplepopulationandcommunity/populationandmigration/populationestimates/methodologies/midyearpopulationestimatesqmi

[13] Office for National Statistics. Lower layer Super Output Area (2011) to major towns and cities (December 2015) lookup for England and Wales [cited 2021 Jun 29]. https://geoportal.statistics.gov.uk/datasets/dc0b24da0880417abc979c705bce3fde_0/explore

[14] Data.ac.uk. UK learning providers [cited 2021 Jun 29]. http://learning-provider.data.ac.uk

[15] COVID outbreak: Manchester Metropolitan University students in lockdown. BBC News [cited 2022 Mar 23]. https://www.bbc.co.uk/news/uk-england-manchester-54289648

[16] Office for Students. Coronavirus briefing note: student accommodation [cited 2022 Mar 23]. https://www.officeforstudents.org.uk/publications/coronavirus-briefing-note-student-accommodation

[17] Vusirikala A, Whitaker H, Jones S, Tessier E, Borrow R, Linley E, et al. Seroprevalence of SARS-CoV-2 antibodies in university students: Cross-sectional study, December 2020, England. J Infect. 2021;83:104–11. 10.1016/j.jinf.2021.04.028 33933527 PMC8081745

[18] Doyle K, Teran RA, Reefhuis J, Kerins JL, Qiu X, Green SJ, et al. Multiple variants of SARS-CoV-2 in a university outbreak after spring break—Chicago, Illinois, March–May 2021. MMWR Morb Mortal Wkly Rep. 2021;70:1195–200. 10.15585/mmwr.mm7035a3 34473687 PMC8422867

[19] Wilson E, Donovan CV, Campbell M, Chai T, Pittman K, Seña AC, et al. Multiple COVID-19 clusters on a university campus—North Carolina, August 2020. MMWR Morb Mortal Wkly Rep. 2020;69:1416–8. 10.15585/mmwr.mm6939e3 33001871 PMC7537562

[20] Cipriano LE, Haddara WMR, Zaric GS, Enns EA. Impact of university re-opening on total community COVID-19 burden. PLoS One. 2021;16:e0255782. 10.1371/journal.pone.0255782 34383796 PMC8360395

[21] Aggarwal D, Warne B, Jahun AS, Hamilton WL, Fieldman T, du Plessis L, et al.; Cambridge Covid-19 testing Centre; University of Cambridge Asymptomatic COVID-19 Screening Programme Consortium; COVID-19 Genomics UK (COG-UK) Consortium. Genomic epidemiology of SARS-CoV-2 in a UK university identifies dynamics of transmission. Nat Commun. 2022;13:751. 10.1038/s41467-021-27942-w 35136068 PMC8826310

[22] Office for National Statistics. Coronavirus (COVID-19) latest insights: deaths [cited 2021 Oct 3]. https://www.ons.gov.uk/peoplepopulationandcommunity/healthandsocialcare/conditionsanddiseases/articles/coronaviruscovid19latestinsights/deaths#deaths-by-age

[23] Cunningham JW, Vaduganathan M, Claggett BL, Jering KS, Bhatt AS, Rosenthal N, et al. Clinical outcomes in young US adults hospitalized with COVID-19. JAMA Intern Med. 2020;181:379–81. 10.1001/jamainternmed.2020.5313 32902580 PMC7489373

[24] The Health Foundation. Generation COVID-19 [cited 2021 Oct 3]. https://www.health.org.uk/publications/long-reads/generation-covid-19

[25] Madewell ZJ, Yang Y, Longini IM Jr, Halloran ME, Dean NE. Household transmission of SARS-CoV-2 a systematic review and meta-analysis. JAMA Netw Open. 2020;3:e2031756. 10.1001/jamanetworkopen.2020.31756 33315116 PMC7737089

[26] Public Health England. COVID-19 vaccine surveillance report week 36 [cited 2021 Sep 14]. https://assets.publishing.service.gov.uk/government/uploads/system/uploads/attachment_data/file/1016465/Vaccine_surveillance_report_-_week_36.pdf

[27] Chudasama DY, Flannagan J, Collin SM, Charlett A, Twohig KA, Lamagni T, et al. Household clustering of SARS-CoV-2 variant of concern B.1.1.7 (VOC-202012-01) in England. J Infect. 2021;83:e26–8. 10.1016/j.jinf.2021.04.029 33933529 PMC8085110

[28] Public Health England. Disparities in the risk and outcomes of COVID-19 [cited 2021 Oct 4]. https://assets.publishing.service.gov.uk/government/uploads/system/uploads/attachment_data/file/908434/Disparities_in_the_risk_and_outcomes_of_COVID_August_2020_update.pdf

