# Supplementary material for: Effect of Returning University Students on COVID-19 Infections in England, 2020
Source: Emerg Infect Dis. 2022 Jul;28(7):1366–74. doi: 10.3201/eid2807.212332 (PMC9239898; doi:10.3201/eid2807.212332)
Supplement: Appendix — Additional information on effect of returning university students on COVID-19 cases in England, 2020. [file 21-2332-Techapp-s1.pdf]

Article DOI: https://doi.org/10.3201/eid2807.212332

Effect of Returning University Students on COVID–19 Cases in England, 2020

Appendix

Appendix Table. Characteristics of included university and non-university towns*

	University towns			Non–University towns		
Region	Town	Population	Pop. density	Town	Population	Pop. density
East of England	Luton	222,588	32.3	Harlow	82,776	31.2
East of England	Colchester	138,533	19.6	Peterborough	180,296	22.5
East of England	Norwich	196,680	29.3	Watford	140,961	26.6
South East	Portsmouth	230,308	35.4	Chatham	78,312	32.6
South East	Reading	259,440	29.1	Hastings	92,661	30.1
South East	Guildford	86,123	29.0	Maidstone	119,704	25.7
West Midlands	Newcastle–under–Lyme	79,766	24.7	Redditch	83,887	25.2
West Midlands	Worcester	103,513	18.7	Solihull	109,827	23.3
Yorkshire & Humber	York	166,470	22.5	Doncaster	116,730	19.5
Yorkshire & Humber	Leeds	511,141	30.5	Grimsby	88,323	30.0

*Pop. density, population per hectare.

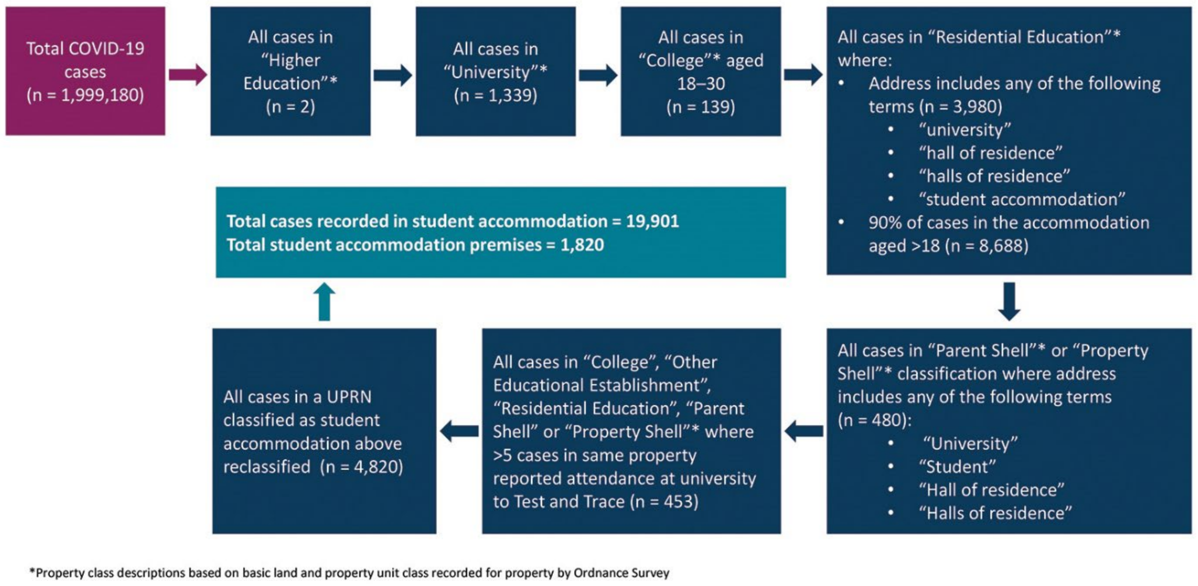

Appendix Figure 1. Classification of properties as student accommodation.

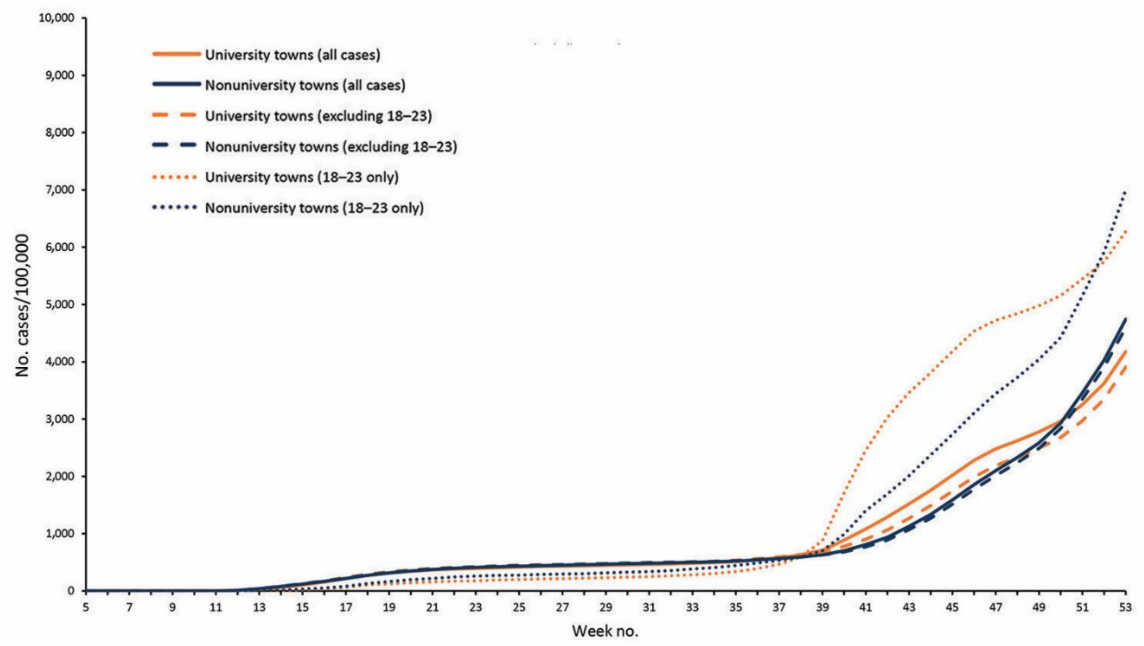

Appendix Figure 2. Cumulative COVID-19 rates in selected university and non-university towns, England 2020.

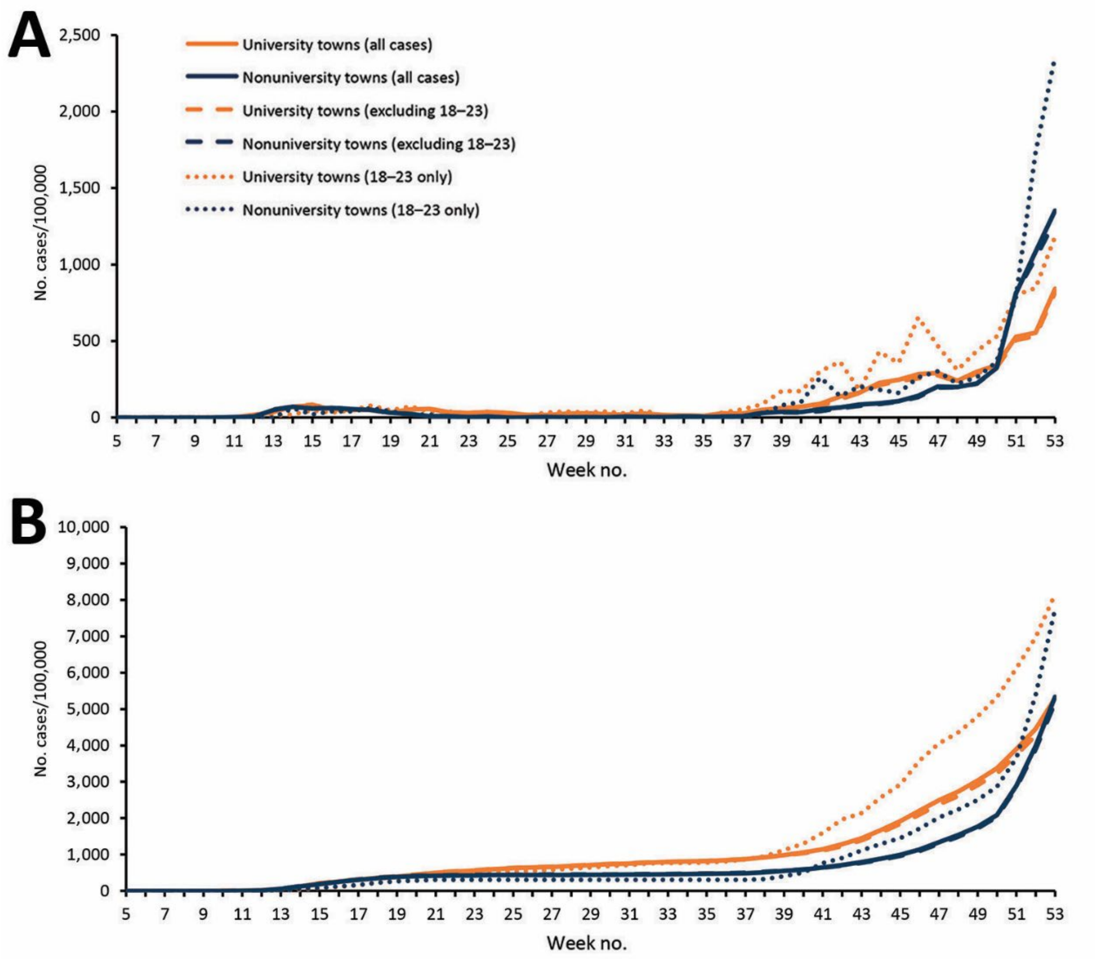

Appendix Figure 3. Weekly and cumulative COVID-19 rates in Luton (university town) (A) and Harlow (non-university town) (B), 2020.

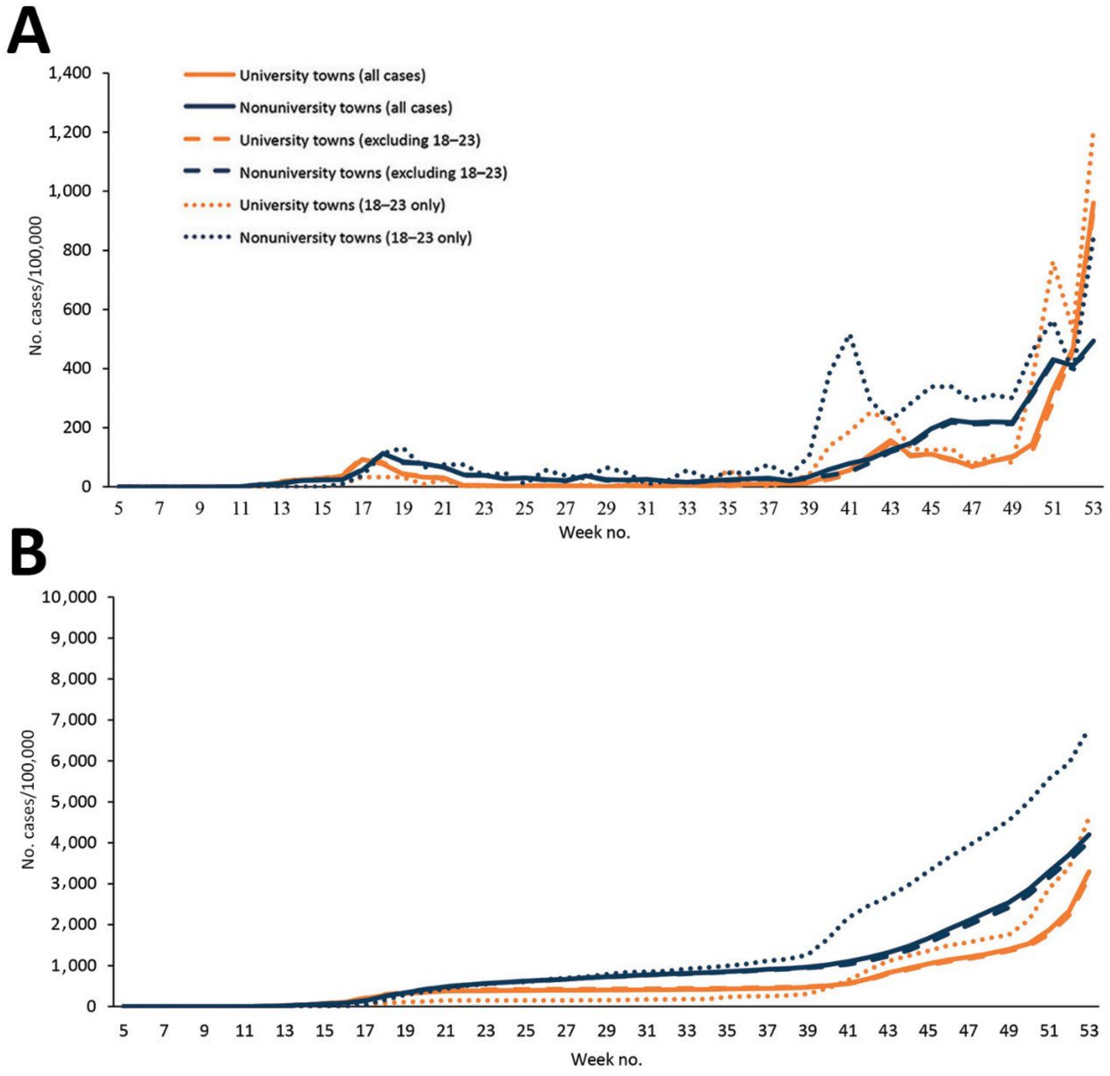

Appendix Figure 4. Weekly and cumulative COVID-19 rates in Colchester (university town) (A) and Peterborough (non-university town) (B), 2020.

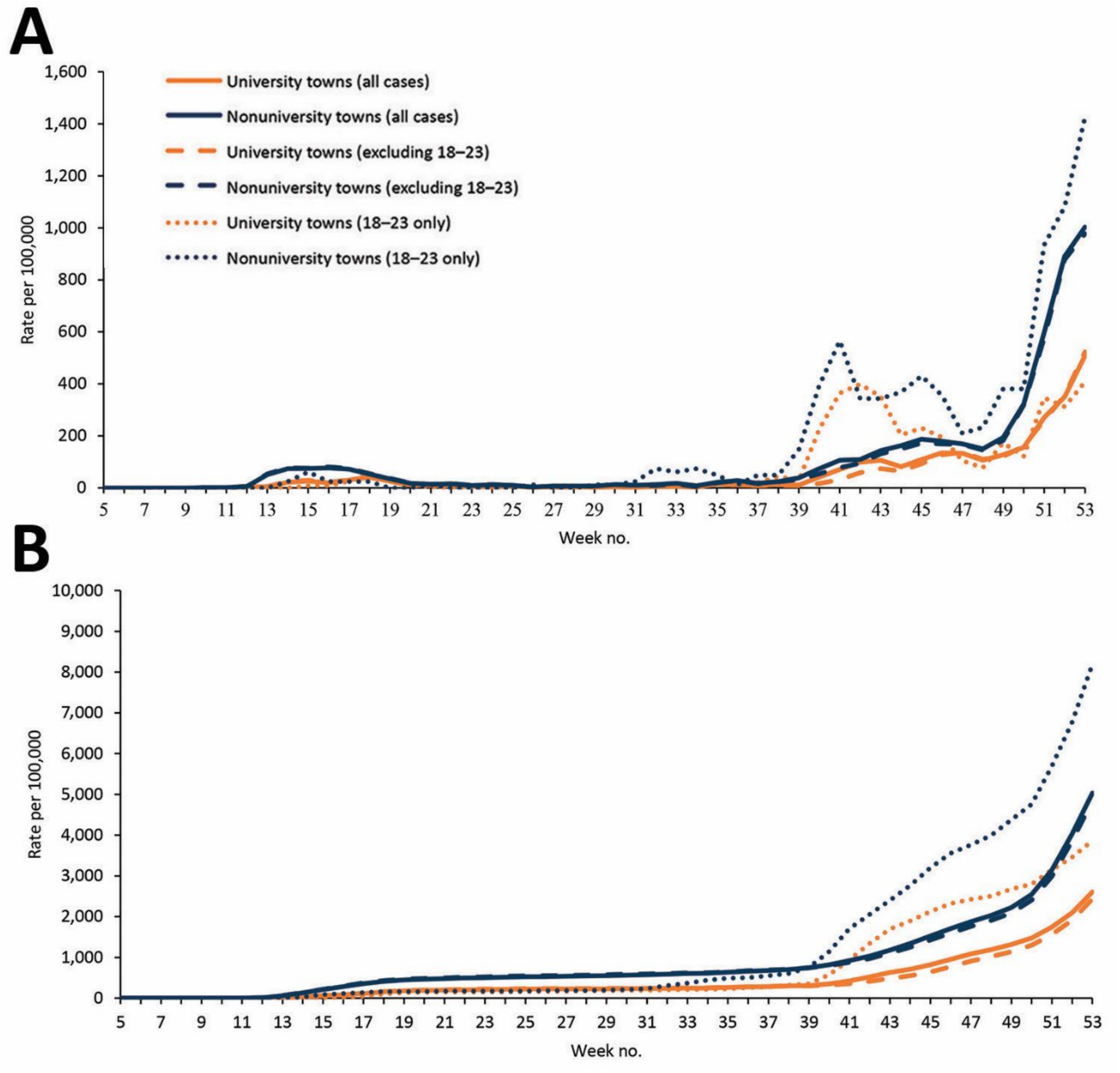

Appendix Figure 5. Weekly and cumulative COVID-19 rates in Norwich (university town) (A) and Watford (non-university town) (B), 2020.

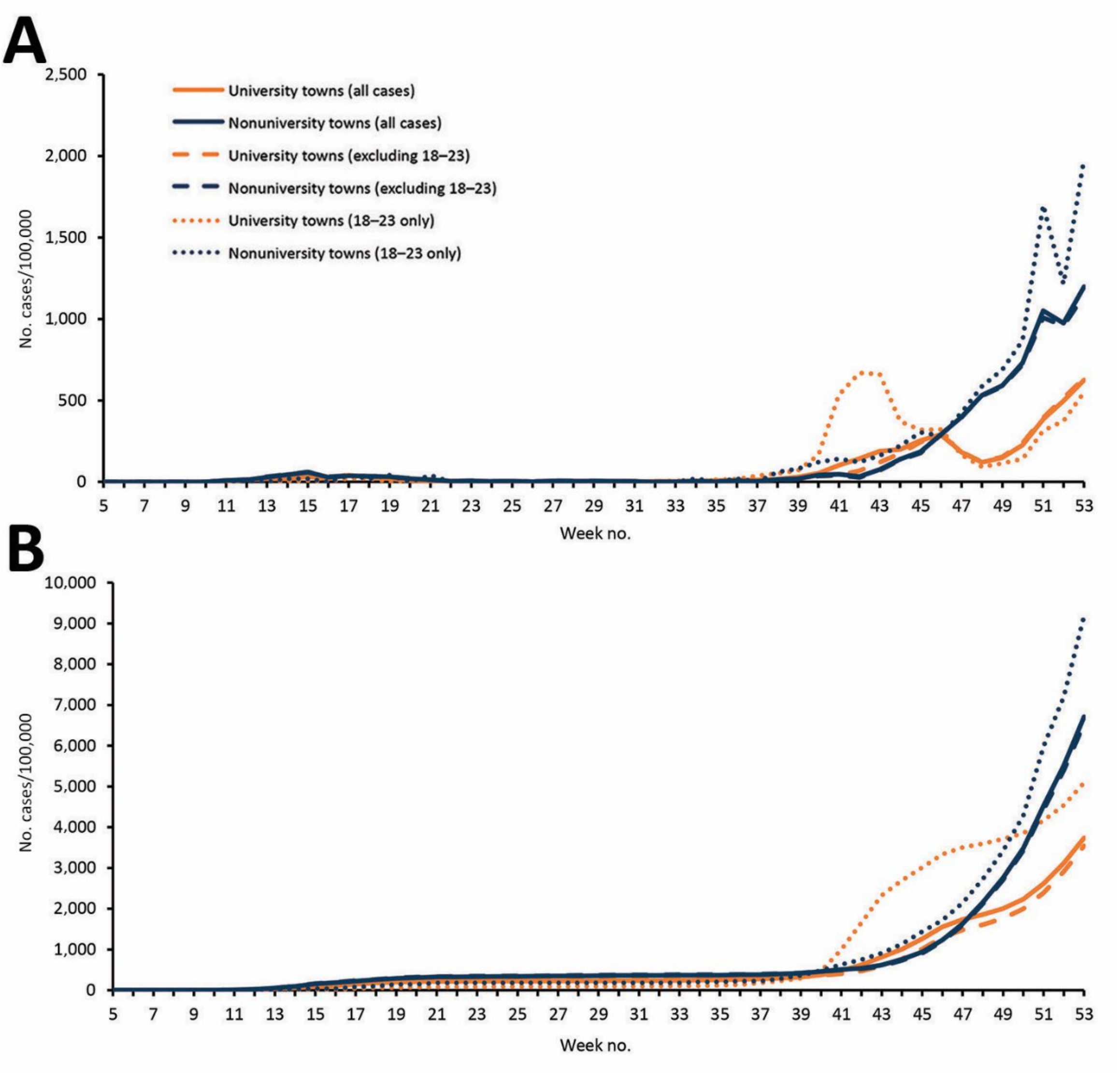

Appendix Figure 6. Weekly and cumulative COVID-19 rates in Portsmouth (university town) (A) and Chatham (non-university town) (B), 2020.

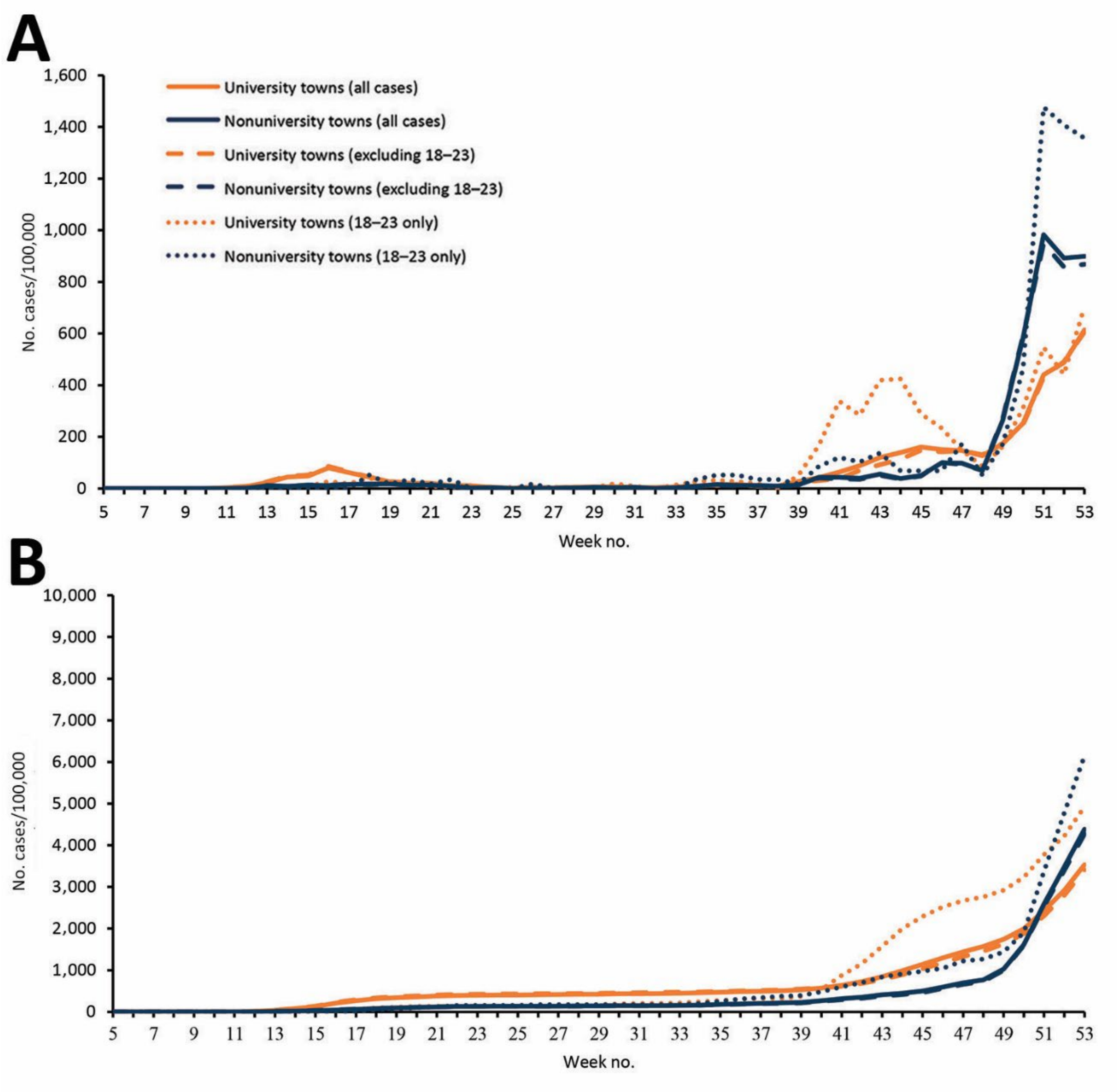

Appendix Figure 7. Weekly and cumulative COVID-19 rates in Reading (university town) (A) and Hastings (non-university town) (B), 2020.

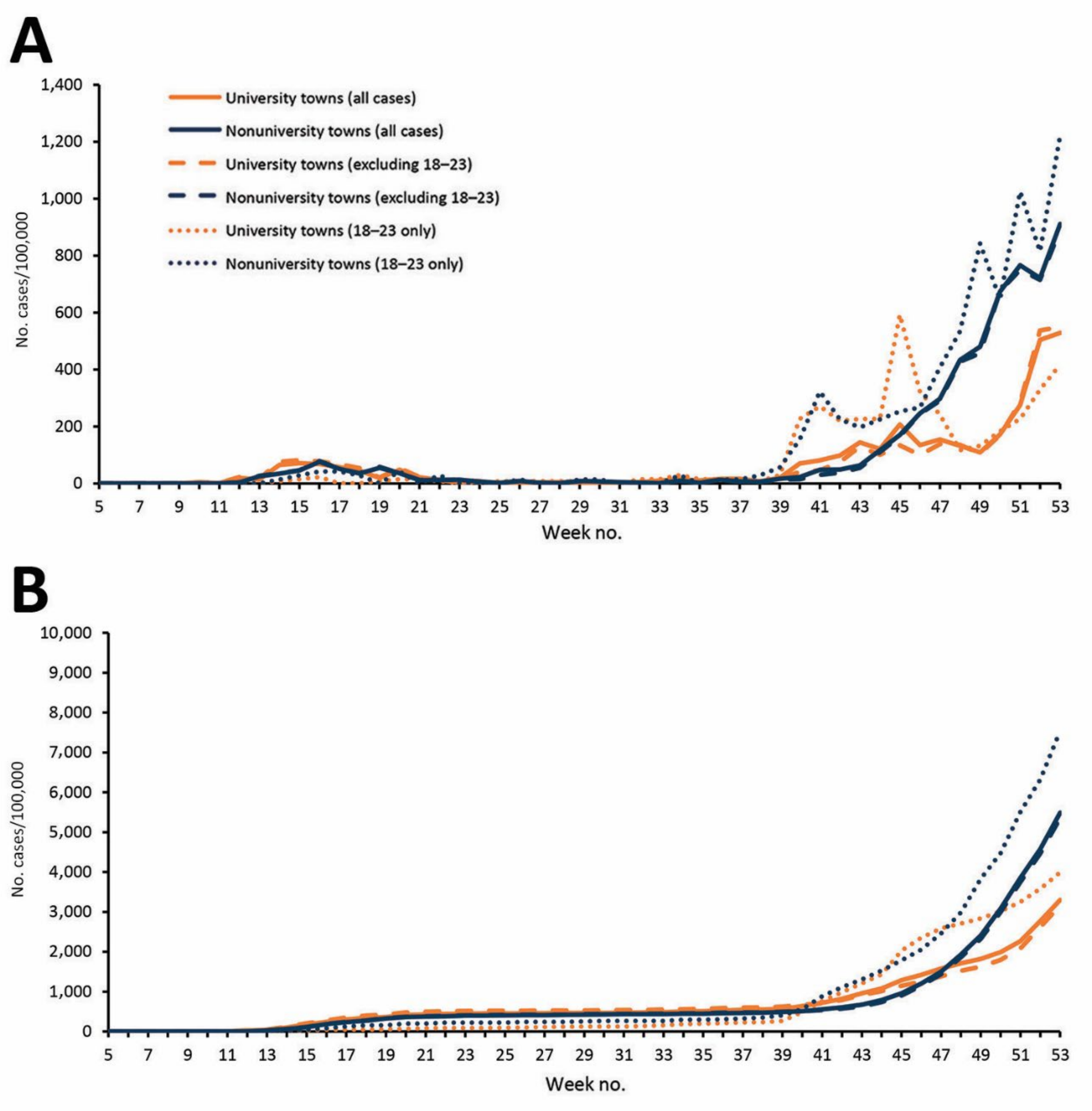

Appendix Figure 8. Weekly and cumulative COVID-19 rates in Guildford (university town) (A) and Maidstone (non-university town) (B), 2020.

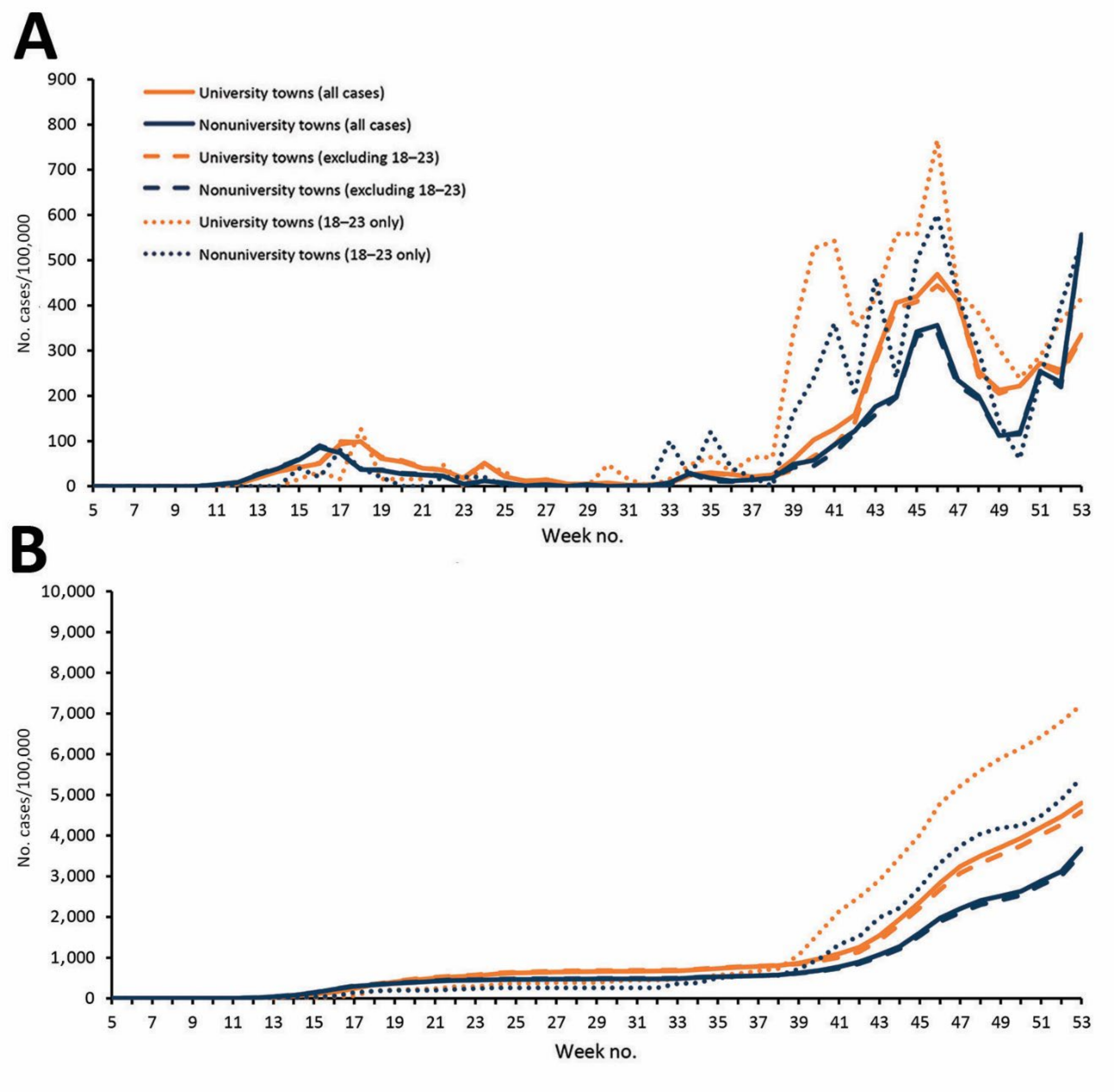

Appendix Figure 9. Weekly and cumulative COVID-19 rates in Newcastle-under-Lyme (university town) (A) and Redditch (non-university town) (B), 2020.

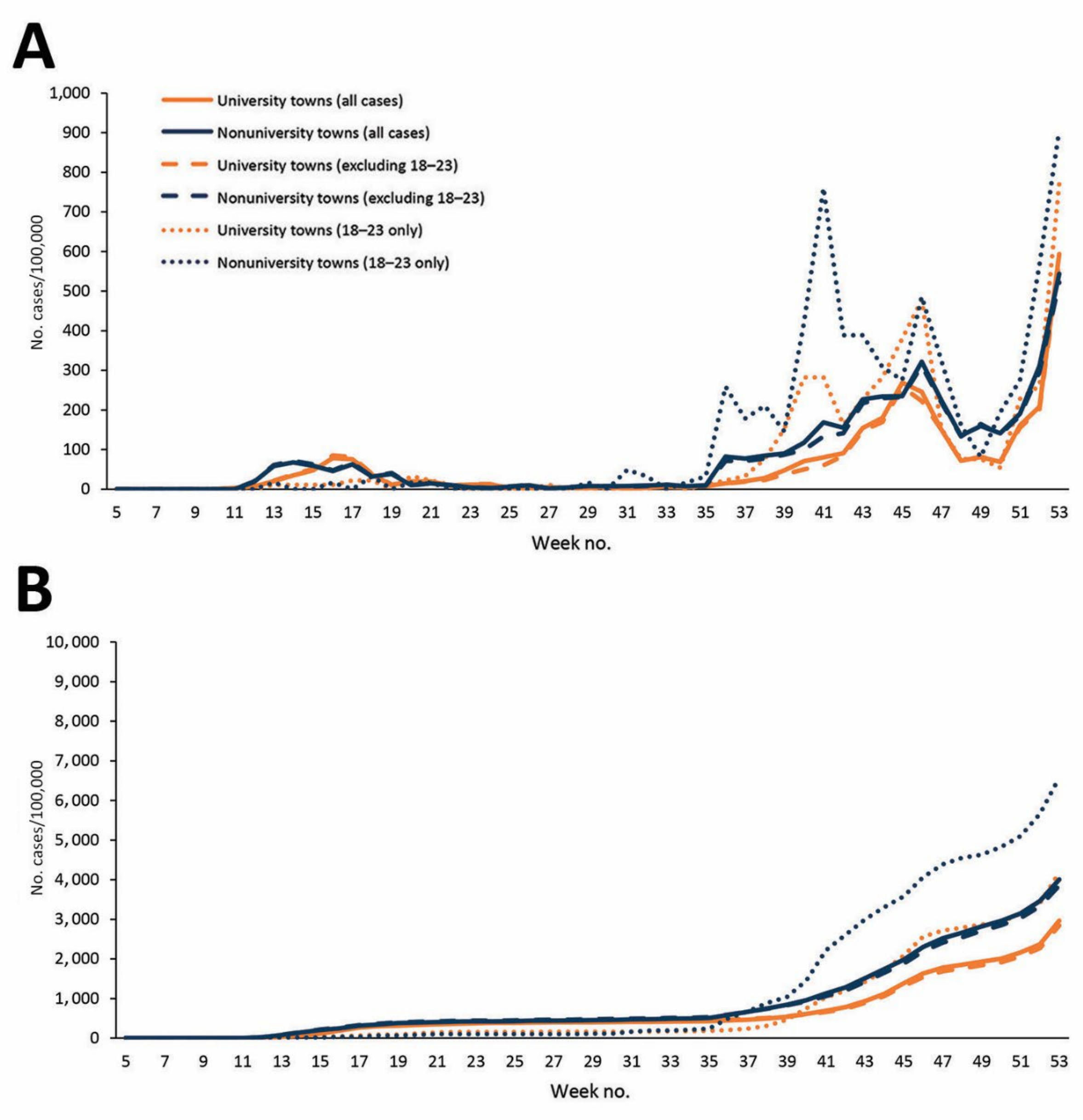

Appendix Figure 10. Weekly and cumulative COVID-19 rates in Worcester (university town) (A) and Solihull (non-university town) (B), 2020.

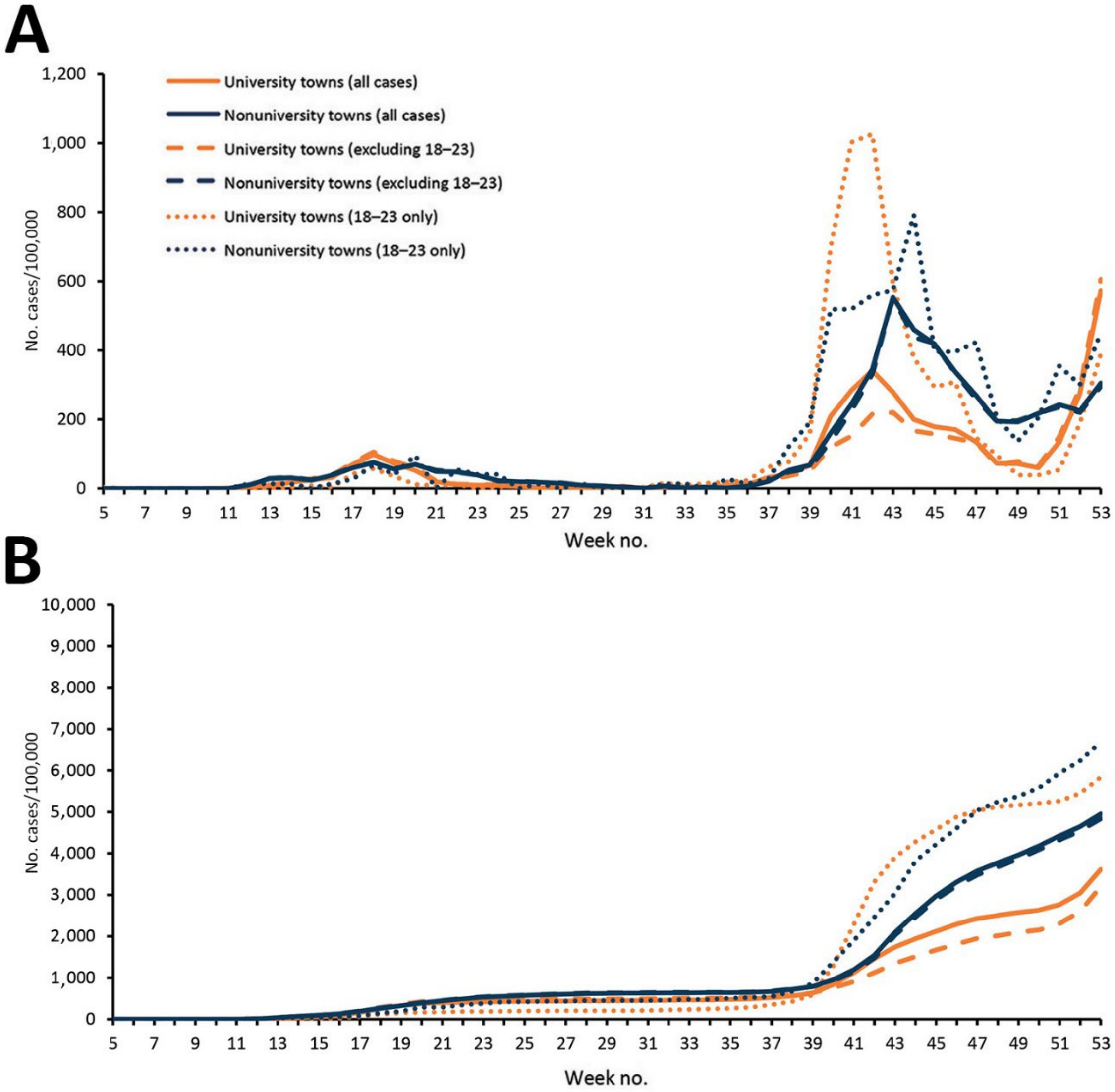

Appendix Figure 11. Weekly and cumulative COVID-19 rates in York (university town) (A) and Doncaster (non-university town) (B), 2020.

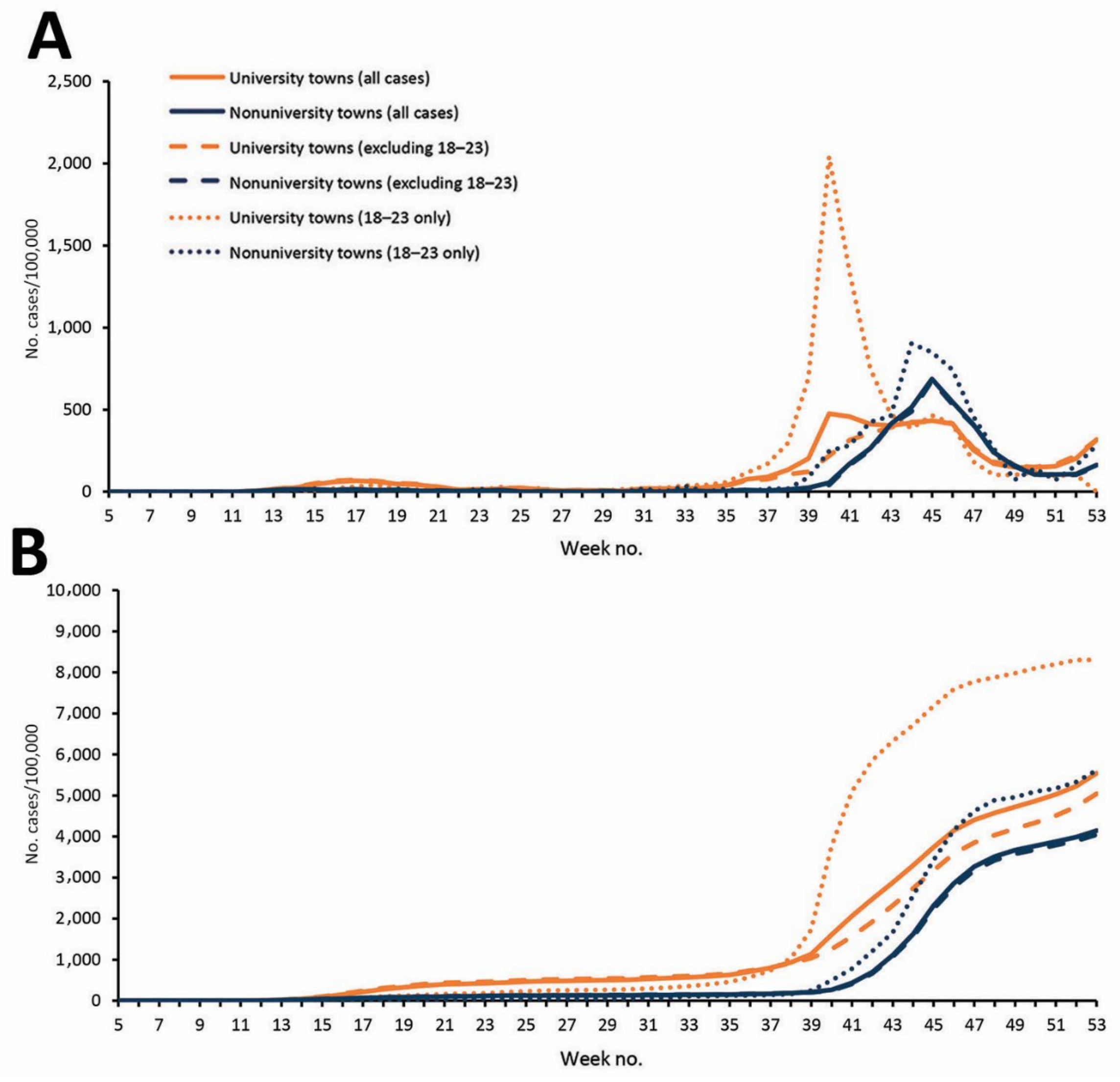

Appendix Figure 12. Weekly and cumulative COVID-19 rates in Leeds (university town) (A) and Grimsby (non-university town) (B), 2020.
